# Improving the Isotretinoin Photostability by Incorporating in Microemulsion Matrix

**DOI:** 10.5402/2011/838016

**Published:** 2011-07-14

**Authors:** Mrunali R. Patel, Rashmin B. Patel, Jolly R. Parikh, Bharat G. Patel

**Affiliations:** ^1^Indukaka Ipcowala College of Pharmacy, New Vallabh Vidyanagar, Anand, Gujarat 388 121, India; ^2^A. R. College of Pharmacy and G. H. Patel Institute of Pharmacy, Vallabh Vidyanagar, Anand, Gujarat 388 120, India; ^3^Institute of Science and Technology for Advance Studies and Research, Vallabh Vidyanagar, Anand, Gujarat 388 120, India

## Abstract

The present paper demonstrates the increased photostability of isotretinoin when loaded in microemulsion. The photodegradation of isotretinoin, in methanol and microemulsion formulation was studied under direct sun light. The photodegradation process was monitored by UV spectrophotometry. In methanol solution, isotretinoin undergoes complete photodegradation just within a few minutes of light exposure. Isotretinoin incorporated in microemulsion formulation showed an increased stability in comparison to the methanol solutions. In particular for isotretinoin, a residual concentration of 75% was still present after a light irradiance versus a residual value of just 16% measured at the same time in methanol solution. Further, degradation kinetic parameters of isotretinoin-loaded microemulsion formulation were demonstrated increase isotretinoin half-life about five-times in comparison with a methanol solution under a direct sun light.

## 1. Introduction

Topical retinoids constitute the core of nearly all therapeutic programs in the mild to moderate acne with an ability to be used to treat noninflammatory and inflammatory acne. The therapeutic success in acne is highly dependent on a regular application of the topical agents over a prolonged period of time. But there are several factors which would seem to indicate that topical application may be problematic. Among them is the extremely low solubility of the compounds, limiting incorporation into an acceptable vehicle, photoinstability which may render topically applied drug ineffective and tolerability problem leading to significant erythema, dryness, peeling, scaling and irritation of skin. This results in discontinuation of treatment or compliance problems in patients who continue treatment [1–3].

Isotretinoin (ITN), a derivative of retinoic acid (13-cis-retinoic acid), is the most effective compound with potential to suppress acne over the long term. However, low solubility of ITN limits its incorporation in a suitable vehicle while its poor photostability renders the topically applied drug ineffective. Therefore, the development of novel formulations characterized by high photoprotection of ITN seems to be important, as well an efficient investigation about the photodegradation processes occurring in the ITN in the developed formulations. The inclusion of the drugs in the novel formulation matrix represents an approach of growing interest to the problem of light-sensitive drugs [4–7].

Novel drug delivery strategies like liposomes, niosomes, aspasomes, microsponges, microemulsion (ME), hydrogels, & solid-lipid nanoparticles can play a pivotal role in optimizing & enhancing the topical delivery of antiacne agents by either modulating their physicochemical & biopharmaceutical properties or minimizing/eliminating the side effects associated with them, thus offering better patient compliance [8–10].

The interest in the development of the drug delivery system for ITN based on ME is because they offer an interesting & potentially a quite powerful way for drug delivery as colloidal drug carrier due to their versatility & attractive advantages. The favourable cutaneous drug delivery properties due to large concentration gradients provided by large drug solubility potential of vehicle affinity for the drug together with ease of formulation, their physical & thermodynamic stability makes ME a very promising vehicles for future topical formulations. Also, topically applied microemulsions (MEs) have been demonstrated to significantly increase the cutaneous absorption of both lipophilic and hydrophilic drugs compared to conventional vehicles for example, aqueous solutions, neat oil phases, micellar solutions, emulsions, and liposomes [11–13].

Drugs photostability represents a significant problem in pharmaceutical research, several articles and reviews dealing with different aspects of such studies are present. Photostability testing for new drugs is included as integral part of stress testing in the ICH guideline [14] and really is an essential activity of the pharmaceutical industry [15]. Formulation and manufacturing process seem to be of decisive importance for drug photostability [16] and several approach systems have been proposed in order to enhance stability for a number of drugs [17].

Photostability studies for retinoic acid formulations have been performed on lotion [18], pharmaceuticals [19], and cosmetics [20]. The inclusion of retinoic acid in liposomes was reported to protect the drug against photodegradation [21], as well as a profound characterization of retinoic acid-liposome [22] and niosome complexes [23] has been described. Shah et al. evaluated the ability of solid-lipid nanoparticle in improving the photostability of tretinoin as compared to tretinoin in methanol and reported that encapsulation of tretinoin in solid-lipid nanoparticle resulted in a significant improvement in its photostability in comparison to methanolic solution and also prevented its isomerization [24]. Ioele et al. reported that tretinoin and ITN undergo a stepwise photodegradation in ethanol with a first very rapid isomerization to 13-*cis *and 9-*cis *isomers, respectively, both followed by a slower degradation to give several isomers. Inclusion of the drugs in liposome matrix was proved to show an improved stability to light. Also, the fast isomerization realized in ethanol from both compounds is avoided in liposomes. They were demonstrated to decrease tretinoin half-life about twelve-times in comparison with an ethanol solution, under a light power of 250 W/m^2^. Further, authors concluded that the inclusion of ITN in liposomes showed a worse performance as compared to tretinoin in liposomes, probably due to low inclusion attributable to its nonlinear molecular structure [25]. 

No literature (to date) was found about the inclusion of ITN in MEs. Hence the present study was undertaken with an aim to increase the photostability of ITN when loaded in ME. ITN-loaded matrixes were subjected to photodegradation studies, either in solution or ME, and an interpretation of the photodegradation process of ITN by determination of kinetic parameters was tried as a complementary aim of the photostability study. Analytical monitoring of the retinoic acids was performed by UV spectrophotometry. 

## 2. Materials and Methods

### 2.1. Apparatus

Absorbance was measured, and spectra were recorded over the wavelength range 200–800 nm in two matched quartz cells with a 1 cm light path using a double beam Perkin Elmer Lambda 19 (Perkin Elmer, Norwalk, CT) UV-Visible spectrophotometer.

### 2.2. Chemicals

ITN was procured as gratis samples from Astron Research Ltd. (Ahmedabad, India). Analytical-grade methanol (E. Merck, Mumbai, India) was used for the sample preparation. Isopropyl Myristate (IPM) was purchased form National Chemicals (Vadodara, India). Caprylocaproyl macrogol-8-glyceride and polyglyceryl oleate were procured as gratis sample form Gattefosse (Saint-Priest, France). Double distilled water was used for the preparation of MEs to avoid surface active impurities. 

### 2.3. Laboratory Precautions

To minimize drugs photodegradation, all handling of ITN was carried out under red lamp (60 W) and whenever possible amberized glassware was used.

### 2.4. Preparation of Standard Stock Solutions

Stock solution was prepared by weighing ITN pure powder (10 mg), weighed powder was accurately transferred to a volumetric flask of 100 mL and dissolved in and diluted to the mark with methanol to obtain a standard stock solution of ITN (100 *μ*g/mL). Working standards, to obtain concentrations within a range of 5.0–40.0 *μ*g/mL for ITN, were prepared by appropriate dilution with methanol of the stock solutions and used to set up the calibration curves.

### 2.5. Preparation of ITN-Loaded Microemulsions

In order to find out the concentration range of components for the existing range of MEs, pseudoternary phase diagrams were constructed using water titration method at ambient temperature. Three phase diagrams were prepared with the 1 : 1, 2 : 1, and 3 : 1 weight ratios of caprylocaproyl macrogol-8-glyceride to polyglyceryl oleate, respectively. For each phase diagram at a specific surfactant (S)/cosurfactant (CoS) mixing ratio (Km), the ratios of oil to the mixture of S/CoS were varied as 0.5 : 9.5, 1 : 9, 1.5 : 8.5, 2 : 8, 2.5 : 7.5, 3 : 7, 3.5 : 6.5, 4 : 6, 4.5 : 5.5, 5 : 5, 5.5 : 4.5, 6 : 4, 6.5 : 3.5, 7 : 3, 7.5 : 2.5, 8 : 2, 8.5 : 1.5, 9 : 1, and 9.5 : 0.5. The mixtures of oil and S/CoS at certain weight ratios were diluted with water dropwise, under moderate magnetic stirring. After being equilibrated at ambient temperature for 24 hours, the mixtures were assessed visually and determined as being MEs, crude emulsions, or ME gels. The stable MEs were also observed under polarizing light to conform their isotropic nature. No attempt was made to distinguish between oil-in-water, water-in-oil, or bicontinuous type MEs. Gels were claimed for those clear and highly viscous mixtures that did not show a change in the meniscus after being tilted to an angle of 90°.

ME formulation was selected from the pseudoternary phase diagram with 3 : 1 weight ratio of caprylocaproyl macrogol-8-glyceride to polyglyceryl oleate. ITN was added to the mixtures of oil and S/CoS, and then an appropriate amount of distilled water was added to the mixture drop by drop and the ME containing ITN was obtained by stirring the mixtures at ambient temperature which was stored at ambient temperature.  Table 1 shows composition of selected ME formulation.

### 2.6. Characterization of ITN-Loaded Microemulsion

The average droplet size and polydispersity index of ME was measured by photon correlation spectroscopy (PCS) with in-built Zetasizer (Nano ZS, Malvern Instruments, UK) at 633 nm. Helium neon gas laser having intensity of 4 mW, was the light source. The droplet size was calculated using Stokes—Einstein relationship by Zetasizer Software. Transmission electron microscopy (TEM) was used to characterize the microstructure of ITN loaded ME. ME was placed on a carbon-coated copper grid and then a drop of 1% phosphotungstic acid covered on MEs. The superfluous phosphotungstic acid on MEs was wiped off by filter paper. The TEM images were obtained using a Tecnai G2 20 TEM (Philips, Holland). The refractive index of the system was measured by an Abbe refractometer (Bausch and Lomb Optical Company, Rochester, NY) by placing 1 drop of solution on the slide. The percent transmittance of the system was measured using a colorimeter (Digital Colorimeter, D-801, Photocon) at 570–590 nm. In order to verify the isotropic nature of ME, samples were examined using cross-polarized light microscopy (Polarizing Microscope, Carlzeless, Jena, Germany). A drop of sample was placed between a cover slip and a glass slide and then observed under cross-polarized light. The pH values of ME was determined using digital pH meter (Orion pH meter 420A, Allometric Ltd., Baton Rouge, LA), standardized using pH 4 and 7 buffers before use. The viscosity of ME was measured using a Brookfield Viscometer (Brookfield Engineering LABS, Stoughton, MA) with spindle LV-III at 100 rpm using interval of 30 seconds. All aspects of testing were controlled using optional Rheocalc Software. The electric conductivity of ME was measured with a conductivity meter (Equip-Tronics, EQ-664, Mumbai, India) equipped with in-built magnetic stirrer. This was done by using conductivity cell (with a cell constant of 1.0) consisting of two platinum plates separated by desired distance and having liquid between the platinum plate acting as a conductor [12, 13].

### 2.7. Photostability Study of ITN in Solution

The photostability of ITN [24, 25] was assessed by recording its absorption spectra over the wavelength range of 200–500 nm in two matched quartz cells with a 1 cm light path using a double beam UV-Visible spectrophotometer (Perkin Elmer, Lambda 19, Norwalk, CT) at the following conditions: scan speed-slow; time response 1 s; spectral band 1 nm. The radiant power was adjusted to the lower value in the instrumental scale, and the cabinet temperature at 25°C. These gentle experimental conditions were set because of the high sensitivity of the drug to light, allowing so to obtain more accurate control of the photodegradation process. Methanolic solution of ITN (concentration: 20 *μ*g/mL) was exposed to natural sunlight (>20,000 Lux) and the UV spectra of the samples were recorded just after preparation (*t* = 0) and at the following time intervals: 15, 30, 60, 90, 120, 150, 180, 210, 240 min, after suitable dilution with methanol. All sample solutions were filtered through nylon 0.45 *μ*m membrane filters.

### 2.8. Phototstability Study of ITN in Microemulsion

ME formulation was exposed to light under the same experimental conditions described above for solution, and recording the spectra at the same irradiation times. For ME formulation, spectrophotometric measurement was performed by diluting it suitably with methanol. Baseline correction was done using a plain ME dispersion diluted suitably with methanol to nullify any possible absorption arising from the excipients. All sample solutions were filtered through nylon 0.45 *μ*m membrane filters. 

 Sufficient care was taken to maintain similar experimental conditions for both the samples, that is, ITN in methanol and ITN in ME. Consequently, an interpretation of the photodegradation process of ITN by determination of kinetic parameters was tried as a complementary aim of the photostability study.

## 3. Results and Discussion

**Figure 1:**
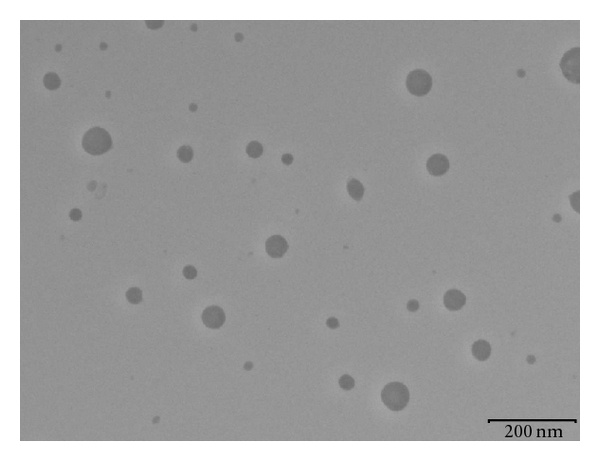
The TEM image of isotretinoin loaded microemulsion containing isopropyl myristate as an oil phase.

Particle size of plain ME and drug-loaded ME were determined, and there was no significant difference observed in average particle size after loading the drug. The ME formulation had the lowest average particle size 45 ± 0.5 nm with polydispersity index (PI) of 0.145 ± 0.027. PI is a measure of particle homogeneity and it varies from 0.0 to 1.0. The closer to zero the PI value the more homogenous are the particles. The PI showed that ME formulation had narrow size distribution. The TEM imaging of ITN loaded MEs is shown in Figure 1. The particle size of ITN loaded MEs from TEM images (Figure 1) accords with that of from PCS. The imaging showed that ITN loaded ME exhibited a spherical shape and had a narrow size distribution. The refractive index of the developed ME was found to be 1.329 and percent transmittance >99% which proved the transparency of the system. The sample was examined by ocular inspection in a cross polarizer for sample homogeneity and birefringence. The ME appeared completely dark when observed under cross polarizer which indicated that it was optically isotropic. The ME formulation had appropriate observed pH value (5.9 ± 0.24) for topical application. Incorporation of ITN did not significantly affect the observed pH value of the ME formulations. The developed system had the low viscosity (31.54 ± 0.23 mPa·s) and high conductivity (149.5 ± 2.21 *μ*S/cm). There was no significant difference found between the viscosities of plain and drug-loaded MEs. The investigated ME formulation containing nonionic surfactant mixture, oil, and water showed electroconductive behaviour in spite of its non-ionic nature. From the viscosity and electroconductive study it could be concluded the prepared ME formulation was of o/w type [12, 13]. 

### 3.1. UV Spectra of ITN

**Figure 2:**
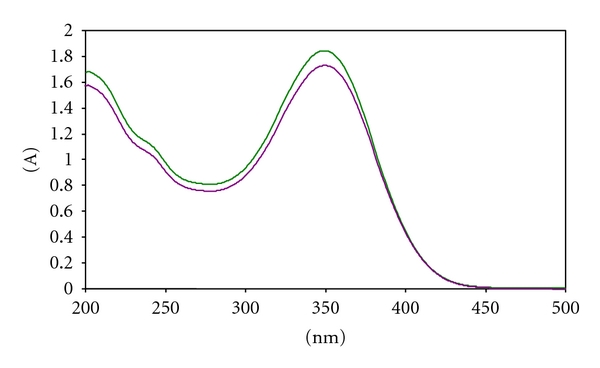
Overlaid UV spectra of 20 *μ*g/mL ITN in methanol solution and 25 *μ*g/mL ITN in microemulsion.

Figure 2 shows the UV spectra of ITN in methanol solutions. As evident, ITN is characterized by the presence of one imposing absorbance maximum at 348 nm. Calibration graphs were obtained by applying least squares regression analysis to the absorbance amplitudes at the single-maximum peaks against the increasing concentrations of pure ITN. The calibration curves parameters are summarized in Table 2.

### 3.2. Photostability of ITN in Solution

**Figure 3:**
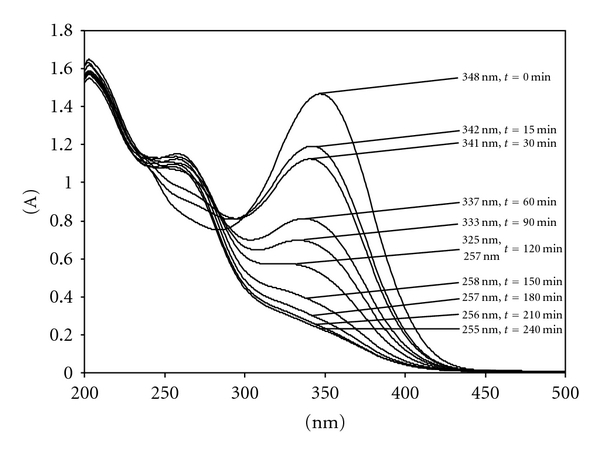
UV spectra of a 20 *μ*g/mL ITN in methanol solution after different times of light exposure.

The spectral curves recorded on the methanolic solution of ITN at the various times of light exposure are shown in Figure 3. The light exposure caused a sharp degradation after 15 minutes of irradiation with a contemporary shift of maximum peak from 348 to 342 nm indicating the isomerization of ITN to *9-cis* isomer [25]. A slower degradation was observed later on with the minimization of the 337 nm maximum peak with a 50% absorbance lowering, compared to the initial value. Further light exposure caused the minimization of this peak with its simultaneous shift to lower wavelengths, probably due to a complex mixture of several retinoic acid isomers. Such results point out the very high sensitivity to light of ITN, suggesting the importance of the development of a pharmaceutical carrier able to minimize its photodegradation. Recent studies have demonstrated the positive action of the novel formulation matrix as carrier systems for a number of photosensitive drugs [19–25]. The first goal of the present study was to investigate the photostabilization of the ITN when loaded in ME through data obtained under conditions of accelerated irradiation. Consequently, an interpretation of the photodegradation process of ITN drug was tried as a complementary study.

### 3.3. Photostability of ITN in Microemulsion

**Figure 4:**
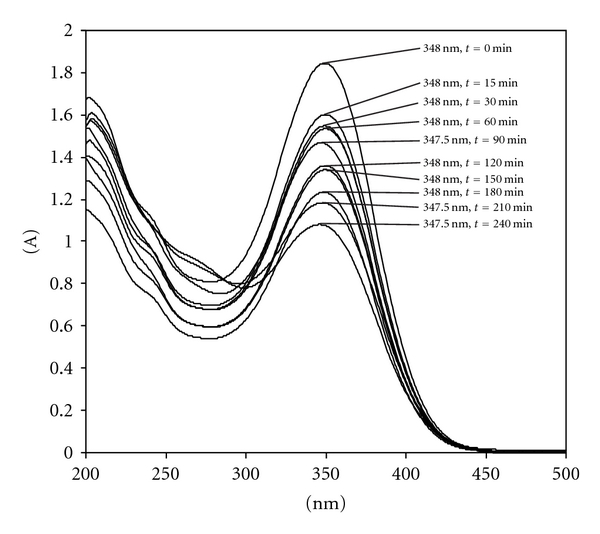
UV spectra of a 25 *μ*g/mL ITN in microemulsion after different times of light exposure.

The photostability studies were carried out by spectrophotometric measurements performed on the ITN loaded ME formulation just before exposure and exposure at increasing times by diluting the samples suitably with methanol.  Figure 4 shows the spectral curves of the ITN loaded ME formulation obtained after the sequential exposure times. As can be seen, the ITN in ME carrier, strongly reduced the photodegradation process as compared to that of the methanolic solution. On the other hand, the photostability of the ITN in ME formulation improved as compared to that recorded of the methanolic solution. Furthermore, significant shift of the maximum peak at 348 nm to lower wavelength was not observed. This may be considered as an interesting result, as it demonstrates no direct isomerization of ITN to 9-*cis *isomers [25]. Also, a residual ITN concentration of 75% was still present after irradiation for and 240 min which was significantly higher in comparison to ITN concentration in methanolic solution at the end of 240 min (*P* < 0.05). The dramatic improvement in the photostability of ITN indicates positive action of the ME matrix as a carrier system. 

In order to evaluate photostability of ME excipients, a parallel analytical control on the formulation matrix photostability was also performed. For this purpose, sample of plain ME was irradiated under the same experimental conditions adopted for the ITN loaded ME. No appreciable changes between the spectra recorded before and after light exposure were detected, clearly showing that any photodegradation occurring during experimental time was negligible.

Ioele et al. reported that the inclusion of ITN in liposomes did not give satisfactory results and shows higher degradation rate than the retinoic acid in liposomes complex studied, but showing an increased stability if compared with the ITN in ethanol solution and concluded that ITN in liposomes showed a worse performance among the drugs studied, probably due to a low inclusion as a result of non-linear molecular structure [25]. In current study, a residual ITN concentration of 75% was found to be present after irradiation for 240 min in ME. On the contrary, in previous study [25] the inclusion of ITN in liposomes did not give analogous satisfactory results, residual ITN concentration was less than 65%.

In a nutshell, we can conclude that ME exhibits a good potential to improve the photostability of ITN and can be employed as a valuable drug delivery strategy.

### 3.4. Kinetic Parameters Determination

The photochemical reaction in methanol solution was demonstrated to cause a very rapid isomerization of ITN followed by a further degradation consisting in the minimization of the absorbance peaks. Therefore, the absorbance values of these maxima were used to evaluate the kinetics of the photodegradation processes [25]. ITN was shown to follow a first order kinetics and a good linearity was obtained by plotting the logarithm of absorbances as a function of time, both in methanolic solution and in ME, in accordance with the following equation:

(1) log⁡  (%A)=−kt+2

where, %*A* was percent residual absorbance, *k *was the photodegradation rate constant, *t *was the time (min), and 2 was the logarithm of initial percent absorbance (100%).

**Figure 5:**
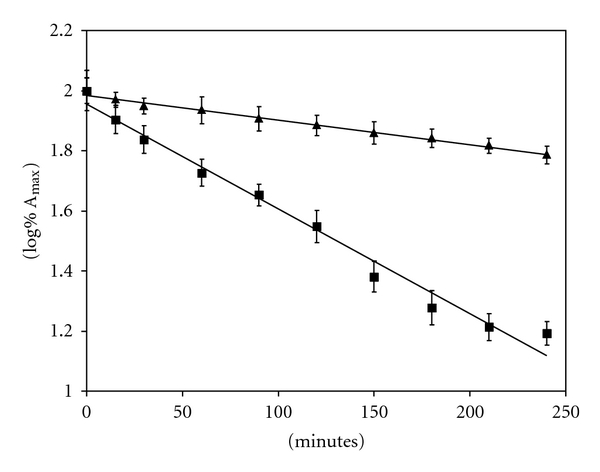
Photodegradation plots of ITN solution in methanol (■) and microemulsion containing ITN (▲). All values are means of three replicated experiments.

The degradation was evaluated on the basis of kinetic photodegradation constant *k *and half-life time (*t*_0.5_), with respect to the initial percent absorbance. ITN degradation curves are plotted in Figure 5 while Table 3 summarizes the degradation kinetic parameters based on data from three replicate analyses for each sample. ME demonstrated to increase ITN half-life about five-times in comparison with a methanol solution, under a direct sun light.

## 4. Conclusion

As it is evident, the ITN inclusion in ME results in a good photostability of the drug, whereas in methanol ITN degraded almost completely after about 240 min under a direct sun light, while in the ME matrix still 75% of residual ITN concentration could be measured. Results obtained from this photostability study lead us to suppose that the better photoprotection of ITN in ME matrix is the consequence of the inclusion of this drug in ME matrix. Further, studied kinetics of the photodegradation processes for ITN loaded ME formulation demonstrated to increase ITN half-life about five-times in comparison with a methanol solution, under a direct sun light. 

The ME system described in the present paper should provide a valuable tool for the development of new pharmaceutical formulations of ITN, capable of improving their photostability.

## Figures and Tables

**Table 1:** Composition of selected microemulsions (%w/w).

Components	Content
Isotretinoin	0.5% w/w
Isopropyl myristate	4.0% wt/wt
Caprylocaproyl macrogol-8-glyceride	31.5% wt/wt
Polyglyceryl oleate	10.5% wt/wt
Aqueous phase	q. s. for 100% wt/wt

**Table 2:** Regression analysis of calibration curve for isotretinoin (ITN) by the proposed UV spectrophotometric method (*n* = 5).

Parameters	ITN
Concentration range	5–40 *μ*g/mL
Slope	0.1115
Standard deviation of the slope	0.57 × 10^−4^
Intercept	−0.1045
Standard deviation of the intercept	0.48 × 10^−3^
Correlation coefficient	0.9998

**Table 3:** Rate constants* of photodegradation for ITN in methanol solution and microemulsion (*n* = 3).

Drug	Sample matrix	*K*	*t*_0.5_	*R* ^2^
ITN	Methanol solution	8.06 × 10^−3^	86	0.9830
	Microemulsion	1.84 × 10^−3^	377	0.9899
	formulation			

*t is expressed in minutes.
